# Dispersion of cardiac action potential duration and the initiation of re-entry: A computational study

**DOI:** 10.1186/1475-925X-4-11

**Published:** 2005-02-18

**Authors:** Richard H Clayton, Arun V Holden

**Affiliations:** 1 Department of Computer Science, University of Sheffield, UK; 2 School of Biomedical Sciences, University of Leeds UK

## Abstract

**Background:**

The initiation of re-entrant cardiac arrhythmias is associated with increased dispersion of repolarisation, but the details are difficult to investigate either experimentally or clinically. We used a computational model of cardiac tissue to study systematically the association between action potential duration (APD) dispersion and susceptibility to re-entry.

**Methods:**

We simulated a 60 × 60 mm 2 D sheet of cardiac ventricular tissue using the Luo-Rudy phase 1 model, with maximal conductance of the K^+ ^channel *gKmax *set to 0.004 mS mm^-2^. Within the central 40 × 40 mm region we introduced square regions with prolonged APD by reducing *gKmax *to between 0.001 and 0.003 mS mm^-2^. We varied (i) the spatial scale of these regions, (ii) the magnitude of *gKmax *in these regions, and (iii) cell-to-cell coupling.

**Results:**

Changing spatial scale from 5 to 20 mm increased APD dispersion from 49 to 102 ms, and the susceptible window from 31 to 86 ms. Decreasing *gKmax *in regions with prolonged APD from 0.003 to 0.001 mS mm^-2 ^increased APD dispersion from 22 to 70 ms, and the susceptible window from <1 to 56 ms. Decreasing cell-to-cell coupling by changing the diffusion coefficient from 0.2 to 0.05 mm^2 ^ms^-1 ^increased APD dispersion from 57 to 88 ms, and increased the susceptible window from 41 to 74 ms.

**Conclusion:**

We found a close association between increased APD dispersion and susceptibility to re-entrant arrhythmias, when APD dispersion is increased by larger spatial scale of heterogeneity, greater electrophysiological heterogeneity, and weaker cell-to-cell coupling.

## 1. Background

Cardiac disease remains an important cause of sudden death in the industrialised world, and in many cases the lethal events are the cardiac arrhythmias called ventricular tachycardia (VT) and ventricular fibrillation (VF). Spontaneous episodes of VT and VF occur in patients where cardiac disease or congenital abnormality has remodelled either the structure or function of cardiac cells and tissue. There is abundant experimental evidence to support the idea that VT and VF are sustained by re-entry [1,2], but the initiation of re-entry in a particular individual is not well understood, and so is difficult to either predict or prevent.

Slow conduction and unidirectional block have long been known to facilitate re-entry [3], and experimental studies have established a link between regional differences in repolarisation, and an increased vulnerability to re-entrant arrhythmias following one or more premature stimuli [4-7]. One of the earliest computer models of activation in cardiac tissue was used to demonstrate that regional differences in repolarisation can allow fibrillation to develop following a premature stimulus [8]. Further experimental studies have found that steep gradients in repolarisation correlate with arcs of conduction block around which re-entry circulates [9-12], and have suggested that regions with longer refractory period must be of a critical size for sustained re-entry to occur [13]. Computational and theoretical studies [14-16] have also shown how a region with prolonged repolarisation can block a premature excitation resulting in initiation of re-entry, and that the size of the inhomogeneity determines the characteristics and persistence of re-entry.

Regional differences in repolarisation are often described as action potential duration (APD) dispersion. The difference between the longest and shortest observed APD is a conceptually simple and easily obtained quantity and has widely been used to measure APD dispersion, although other indices have been proposed [17]. Some experimental studies have established critical values of APD dispersion above which re-entry is initiated consistently [18]. In others the gradient of APD has been measured, and spatial gradients of between 2 and 12.5 ms mm^-1 ^were associated with block and re-entry [9,19,20]. Spatial APD gradients arise from regional differences in ion channel function, but their magnitude depends on electrotonic current flow during repolarisation. APD dispersion can be produced by the spatial scale of regional differences and the magnitude of functional heterogeneity, and is modulated by electrotonic current flow which depends on the strength of cell-to-cell coupling [16,21,22]. The relative effect of these three quantities on APD dispersion and vulnerability to re-entry is important because both disease and congenital abnormalities can result in changes to one or more of them. However, it is difficult to control these tissue properties independently in experiments.

Computational models offer a powerful research tool for addressing these questions, because the properties of a virtual tissue can be controlled precisely and independently in a way that would be extremely difficult to achieve experimentally. The purpose of this study was therefore to investigate systematically how measured APD dispersion and vulnerability to re-entry in a computational model of ventricular tissue are related to: (i) the spatial scale of heterogeneity, (ii) the magnitude of differences in K^+ ^channel conductance between regions with short and long APD, and (iii) strength of cell-to-cell coupling.

## 2. Methods

### 2.1 Computational model of electrical activation

We simulated electrical activation in a 2 D isotropic monodomain virtual tissue [23]



where *V*_*m *_is membrane voltage, *C*_*m *_specific membrane capacitance, D a diffusion coefficient and *I*_*ion *_current flow through the cell membrane per unit area. We used the Luo-Rudy phase 1 (LR1) model [24] to give *I*_*ion*_,



where *I*_*Na*_, *I*_*Ca *_(described as *I*_*si *_in the original model) and *I*_*K *_are time and voltage dependent currents flowing through Na^+^, Ca^2+^, and K^+ ^channels, *I*_*K*1 _a time-independent K^+ ^current, *I*_*Kp *_a plateau K^+ ^current, and *I*_*b *_a background current. We changed two parameters from the original Luo and Rudy paper [24]. We reduced maximum Na^+ ^conductance from 0.23 mS mm^-2 ^to 0.16 mS mm^-2 ^as in the later version of the model [25], and we reduced the maximum conductance of the slow inward current from 0.0009 mS mm^-2 ^to 0.0005 mS mm^-2 ^to produce an APD comparable to that in the canine ventricle. We controlled repolarisation by varying maximum K^+ ^conductance (*gKmax*) from the default value of 0.00282 mS mm^-2 ^to a value between 0.001 mS mm^-2 ^and 0.004 mS mm^-2 ^(see below).

### 2.2 Numerical methods

We solved equation 1 and the LR1 equations using an explicit Euler method, with both a lookup table of the voltage dependent parameters in the LR1 model, and an adaptive operator splitting technique [26]. We applied no-flux boundary conditions, set *C*_*m *_to 0.001 μF mm^-2^, and set D to between 0.05 and 0.2 mm^2 ^ms^-1^. We used an adaptive timestep of either 0.02 or 0.1 ms depending on the magnitude of *dV*_*m*_/*dt *at each grid point [26]. With a space step of 0.2 mm, and D of 0.1 mm^2 ^ms^-1 ^we obtained a conduction velocity (CV) for a stable plane wave of 0.56 m s^-1^, with a speedup of about two times and an error in CV of 2.5 % compared to computations with a fixed timestep of 0.01 ms. Simulations with smaller fixed and adaptive timesteps yielded plane waves with a comparable CV. Changing the space step to 0.25 mm and 0.15 mm resulted in a change of CV for a plane wave of <5 % compared to the CV computed with a space step of 0.2 mm. These findings indicated the stability of our numerical method.

**Figure 1:**
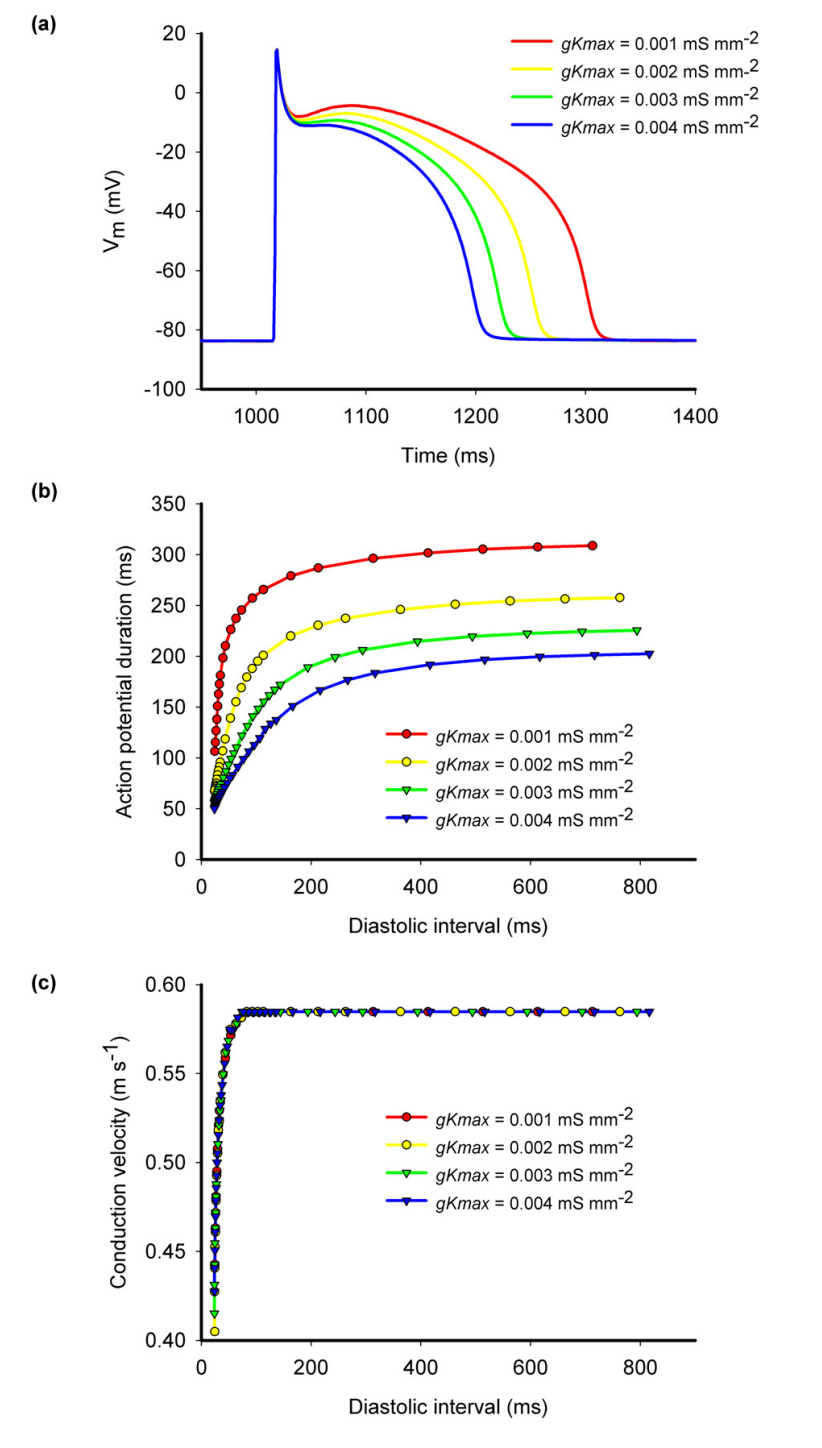
(a) Action potentials recorded during steady pacing at 500 ms intervals for different values of *gKmax*. (b) Action potential duration restitution and (c) conduction velocity restitution for different values of diastolic interval measured with a premature stimulus during steady pacing at 500 ms intervals. All measurements obtained from a narrow strip of virtual tissue 10 mm long and with uniform *gKmax *of 0.001, 0.002, 0.003, and 0.004 mS mm^-2 ^as indicated.

Figure 1 shows action potentials, APD restitution curves, and CV restitution for virtual tissue with different values of *gKmax*. In each case a propagating action potential could be elicited with a minimum diastolic interval of about 10 ms, indicating that the refractory period was very close to the APD.

### 2.3 Virtual tissue and heterogeneity

**Figure 2:**
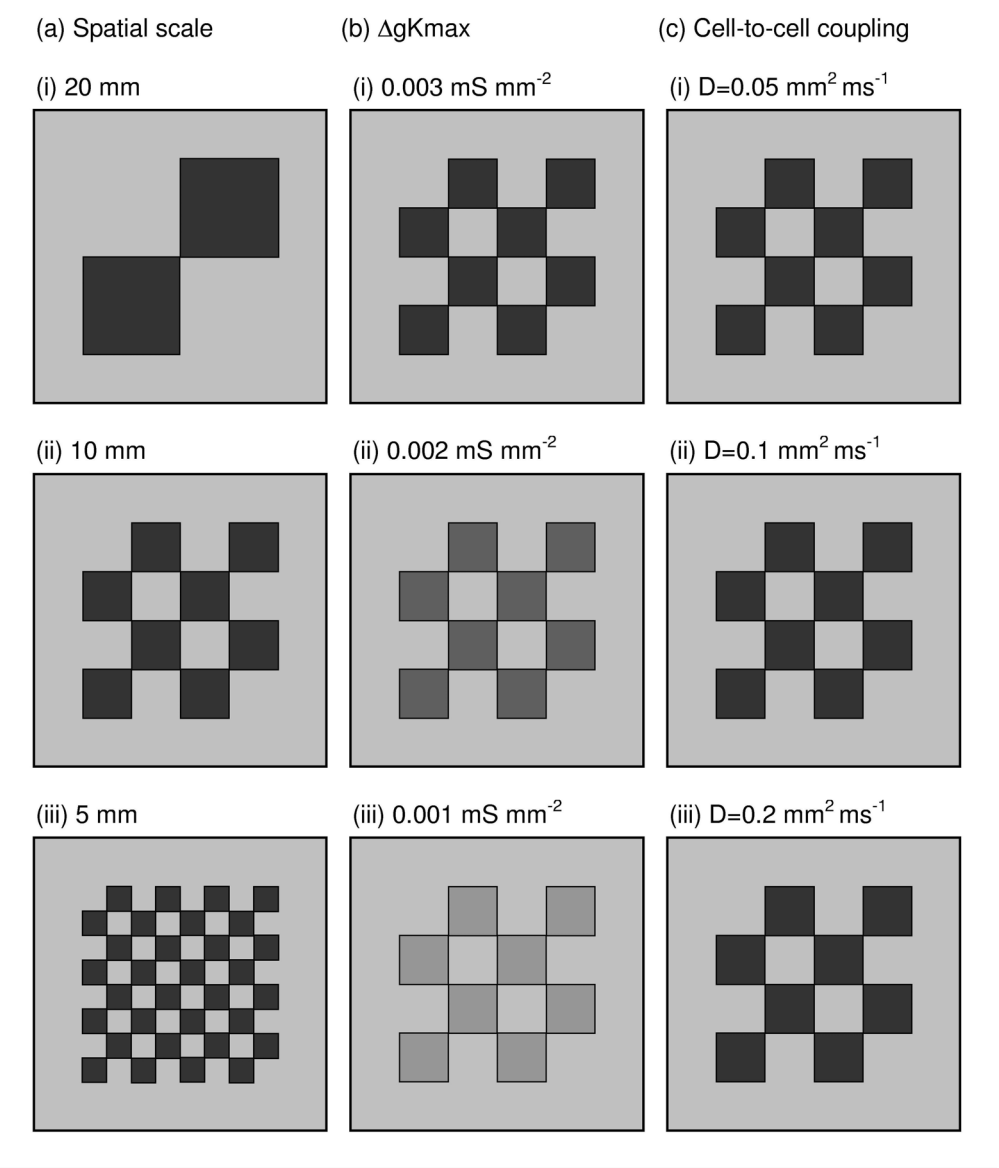
Configuration of 60 × 60 mm virtual tissues used in the study where (a) spatial scale, (b) Δ*gKmax*, and (c) strength of cell-to-cell coupling were varied as indicated on the figure. Greyscale shows *gKmax*, in each region, with light grey, mid grey, dark gray and black corresponding to *gKmax *values of 0.004, 0.003, 0.002, and 0.001 mS mm^-2 ^respectively.

Each of the virtual tissues in this study represented a 60 × 60 mm 2 D sheet with a 10 mm border around each edge with *gKmax *set to 0.004 mS mm^-2^. The central 40 × 40 mm region was heterogeneous, and was subdivided into squares. Within alternate squares *gKmax *was set to either 0.004 mS mm^-2 ^or to a specific lower value (see below). Thus the virtual tissues had a border region with short APD, and a central heterogeneous region divided into a chequerboard with alternating squares of either short or long APD (Figure 2).

In our reference virtual tissue, the 40 × 40 mm heterogeneous region was divided into 16 squares giving heterogeneity with a spatial scale of 10 mm. In half of the 16 squares *gKmax *was set to 0.001 mS mm^-2^, giving a functional heterogeneity with a difference in *gKmax *(Δ*gkmax*) of 0.003 mS mm^-2^. The diffusion coefficient in the whole virtual tissue was set to 0.1 mm^2 ^ms^-1^.

We varied the *spatial scale *of heterogeneity by changing the size of heterogeneity in the reference virtual tissue from 10 mm to 20 mm and 5 mm (Figure 2a), varied Δ*gkmax *from 0.003 to 0.002 and 0.001 mS mm^-2 ^by changing *gKmax *in the alternate squares from 0.001 to 0.002 and 0.003 mS mm^-2 ^respectively (Figure 2b), and varied the strength of *cell-to-cell coupling *by changing the diffusion coefficient from 0.1 to 0.05 and 0.2 mm^2 ^ms^-1 ^(Figure 2c).

### 2.4 APD dispersion

Action potentials were initiated in each virtual tissue by holding the membrane voltage along one edge at 0 mV for 2 ms. We measured APD to 90% recovery (APD_90_) at every grid point. We estimated APD dispersion during steady pacing with three measured that have been used in experimental studies [17]. First we measured APD across the whole virtual tissue, and determined the difference between the maximum and minimum APD (*APDdiff*). Second we measured the standard deviation of APD (*APDSD*) across the whole virtual tissue. Finally we measured the APD difference between each grid point and its neighbours 1 mm above, below, left and right, and determined the maximum value of measurements throughout the whole virtual tissue (*maxLD*) [17].

### 2.5 Vulnerability to re-entry

A spatially homogenous control virtual tissue with uniform *gKmax *supported propagating plane waves following S1 stimulation along one edge. These propagating plane waves had a depolarising wavefront and a repolarising wave back both aligned parallel to the edge that was stimulated. A premature S2 stimulus delivered to the same edge as the S1 stimulus therefore resulted either in block or in a propagating plane wave.

In the heterogeneous virtual tissues the wave back was not a plane wave because some regions repolarised more quickly than others. A premature S2 stimulus could produce a wavefront that would encounter a mixture of recovered and refractory tissue, and hence elicit wavebreak and re-entry. We therefore assessed vulnerability to re-entry in the heterogeneous virtual tissues by delivering two S1 stimuli to one edge at 500 ms intervals, and then a premature S2 stimulus to the same edge. We varied the timing of the S2 stimulus in steps of 1 ms. The virtual tissue response was characterised as either *block *if the S2 stimulus failed to propagate, *re-entry *if the S2 stimulus elicited re-entry that completed more than one cycle, *wavebreak *if the S2 stimulus elicited a wave that broke but did not re-enter, or *propagation *if the S2 stimulus elicited a wave that propagated without wavebreak.

Vulnerability to re-entry is typically estimated by applying a local premature stimulus that interacts with repolarising tissue, and the vulnerable window is the range of stimulus strength and timing that elicits re-entry. In this study, we investigated the initiation of re-entry by S1 S2 stimulation from the same stimulus site, and we estimated the vulnerability of each virtual tissue to re-entry from the range of S2 intervals that resulted in either wavebreak or re-entry. To avoid confusion we refer to our estimate of vulnerability as *susceptibility *to re-entry, and to the range of S2 intervals as the width of the *susceptible *window.

### 2.6 Potential antiarrhythmic strategies

We made a preliminary investigation into two candidate mechanisms for reducing susceptibility, inactivation of the Na^+ ^channel and conductance of the time independent K^+ ^channel *g*_*K*1_.

Block of an action potential occurs if there is insufficient Na^+ ^current to support a propagating wavefront [27]; when Na^+ ^channels have not recovered from inactivation, then a propagating wave blocks and dissipates [28]. Na^+ ^channel inactivation is controlled by the *j*-gate in the LR1 model [24]. We prolonged recovery of Na^+ ^channels from inactivation throughout the virtual tissue by multiplying the time constant of Na^+ ^channel inactivation τ_j _by 10 [29].

The time independent K^+ ^current *i*_*K*1 _is a voltage dependent current that holds the membrane at its resting potential. It is activated during repolarisation and at rest, and is also activated close to the core of re-entrant waves [30,31]. We investigated the effect of doubling the conductance of *i*_*K*1 _throughout the virtual tissue.

## 3. Results

### 3.1 Propagation and APD dispersion during pacing

**Figure 3:**
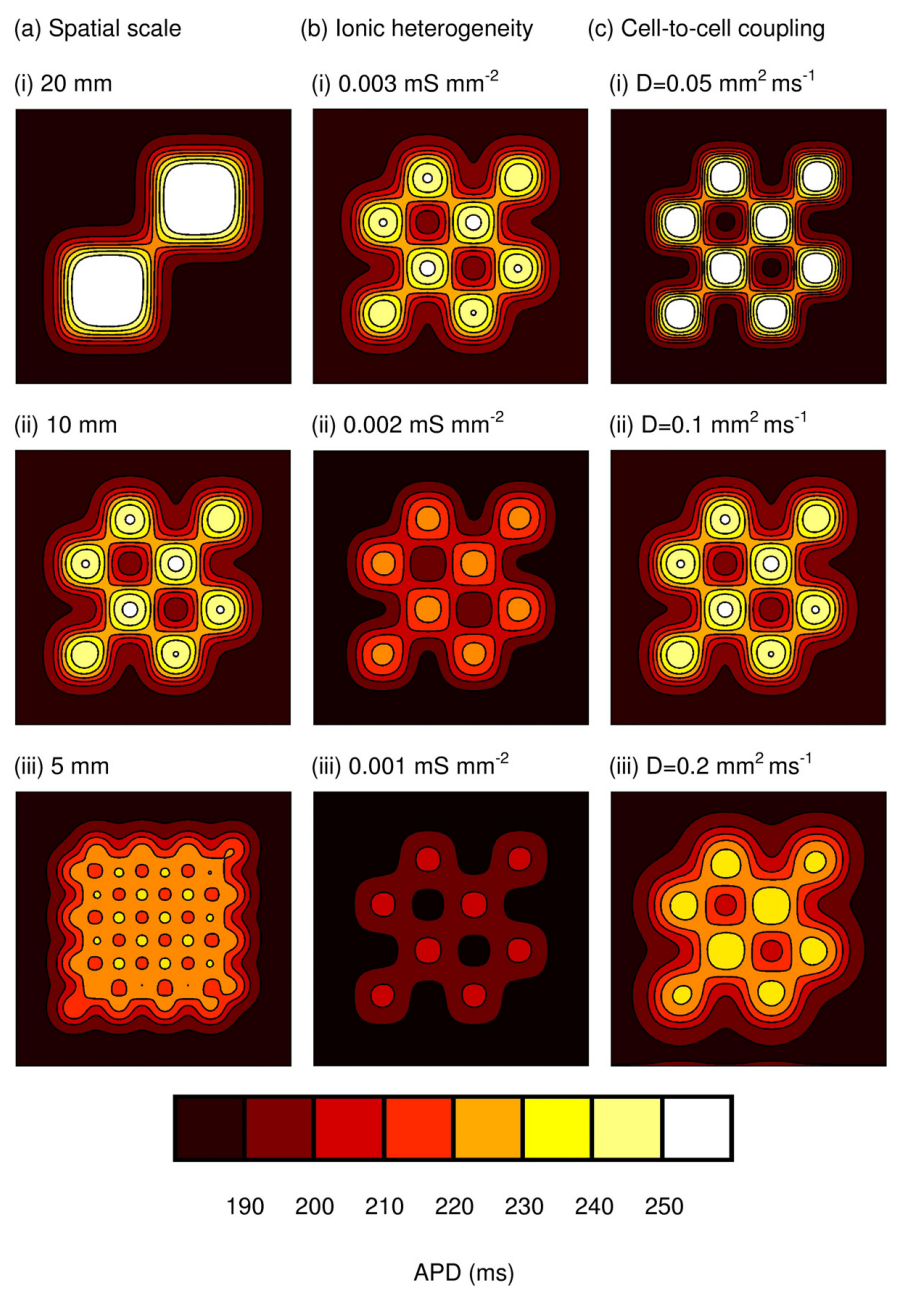
Spatial distribution of APD during from pacing at 500 ms intervals in each variant of the 60 × 60 mm virtual tissue. (a) Effect of changing spatial scale, with Δ*gKmax *fixed at 0.003 mS mm^-2^, and diffusion coefficient fixed at 0.1 mm^2 ^ms^-1^. (b) Effect of changing Δ*gKmax*, with spatial scale fixed at 10 mm, and diffusion coefficient fixed at 0.1 mm^2 ^ms^-1^. (c) Effect of changing strength of cell-to-cell coupling by changing the diffusion coefficient, with spatial scale fixed at 10 mm, and Δ*gKmax *fixed at 0.003 mS mm^-2^.

**Figure 4:**
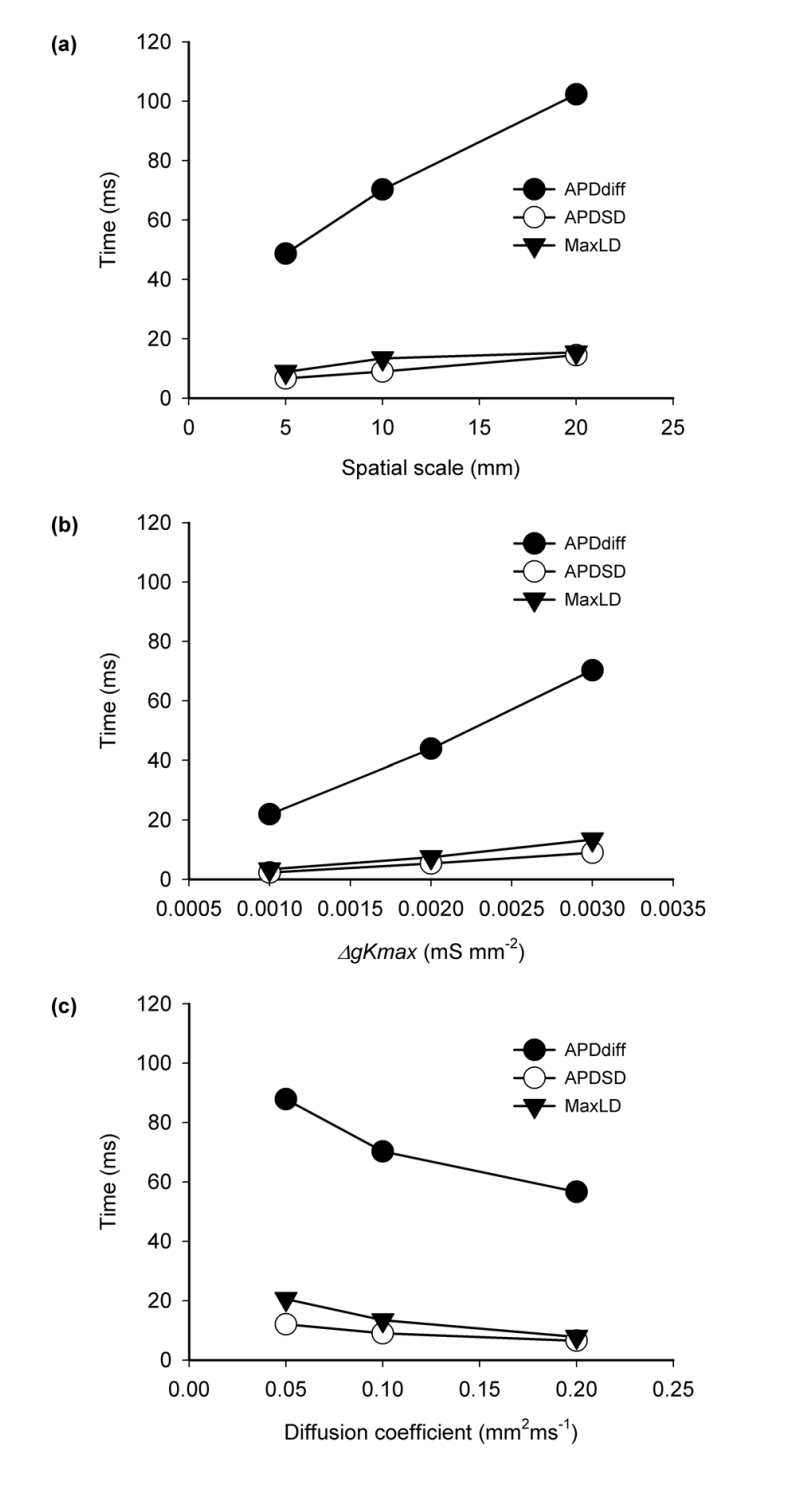
*APDdiff*, *APDSD*, and *MaxLD *measured in virtual tissues where (a) the spatial scale of heterogeneity, (b) magnitude of functional heterogeneity, and (c) strength of cell-to-cell coupling were changed.

Figure 3 shows the spatial distribution of APD_90 _in variants of the virtual tissue during pacing at a cycle length of 500 ms from the bottom edge. The three columns show the effect of changing spatial scales (Figure 3a), Δ*gkmax *(Figure 3b), and strength of cell-to-cell coupling (Figure 3c). The range of colours and number of contours on each figure indicates the range of APD. Hence the leftmost column shows that when Δ*gkmax *and the strength of cell-to-cell coupling are held constant, increasing spatial scale increases the range of APD and decreasing spatial scale decreases the range of APD. Overall, Figure 3 shows that small spatial scales, small Δ*gkmax*, and strong cell-to-cell coupling act to reduce the range of observed APD. This effect can also be seen in Figure 4, which summarises how the three measures of APD dispersion were affected by spatial scale, Δ*gkmax*, and strength of cell-to-cell coupling. Each of these measures changed monotonically within the range of spatial scale, Δ*gkmax *and strength of cell-to-cell coupling that we studied. *APDdiff *was halved by reducing spatial scale from 20 to 5 mm or by increasing the diffusion coefficient from 0.05 to 0.1 mm^2 ^ms^-1^.

When re-entry was initiated, we observed break-up into multiple re-entrant wavelets with up to 18 phase singularities. The mechanism of instability was likely to be a combination of the spatial heterogeneity leading to localised conduction block combined with dynamical instability resulting from steep APD restitution [32] (Figure 1), but was not investigated explicitly. In some simulations the re-entrant waves coalesced and re-entry spontaneously terminated. However, there was no clear association between this observation and S2 timing, spatial scale, functional heterogeneity, or coupling.

### 3.2 Susceptibility to re-entry

**Figure 5:**
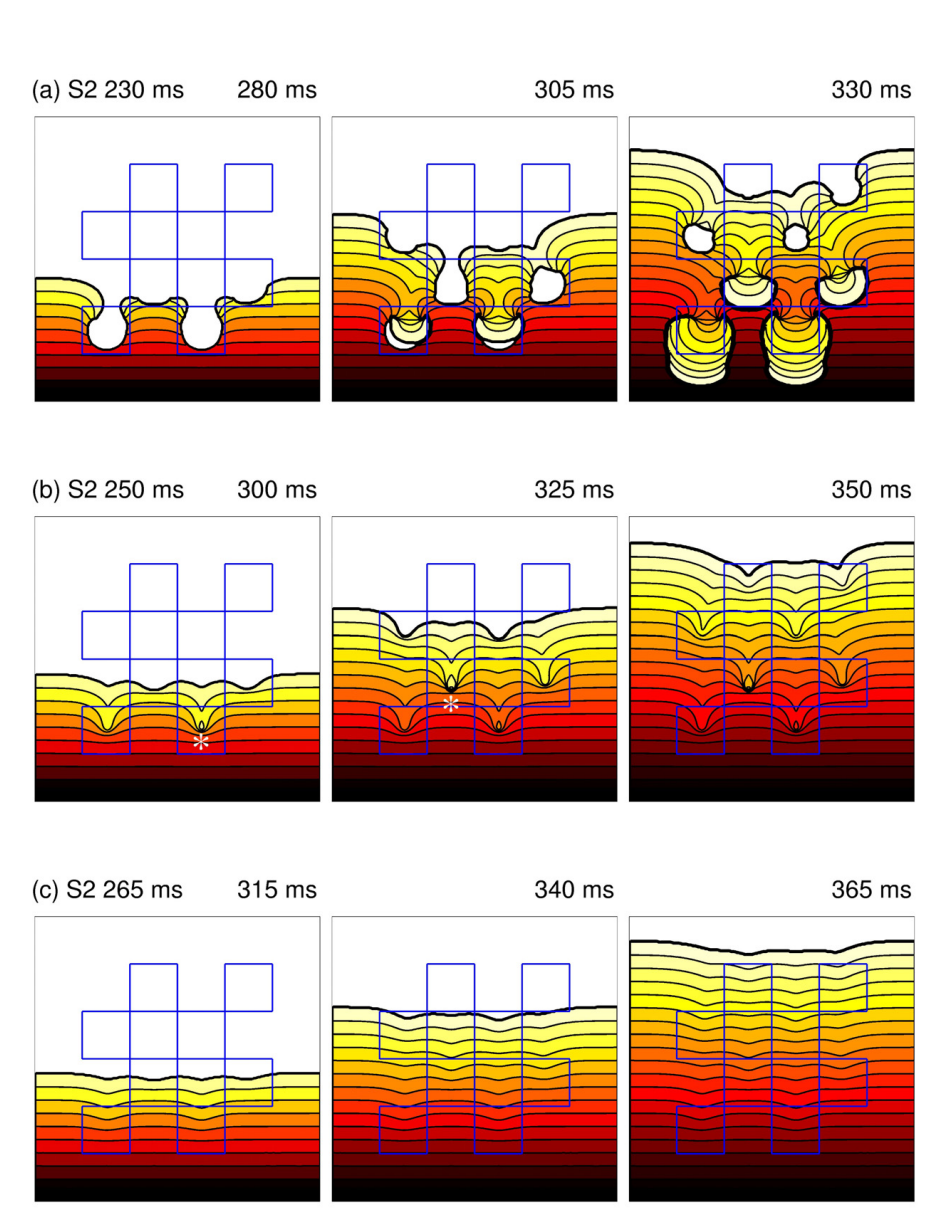
Example responses to premature S2 stimulus in virtual tissue with heterogeneity on spatial scale of 10 mm, Δ*gKmax *of 0.003 mS mm^-2^, and diffusion coefficient set to 0.1 mm^2 ^ms^-1^. Blue lines outline regions with prolonged repolarisation.. (a) S2 at 230 ms and induction of re-entry. (b) S2 at 250 ms, wavebreaks are indicated with a star. (c) S2 at 265 ms and delayed propagation with no wavebreak. In (a-c) snapshots 50, 100 and 150 ms after the S2 stimulus are included, with propagation from bottom to top of the figure. Isochrones at intervals of 5 ms are shown, and colour coding indicates the progress of the wavefront. Movies of the simulations shown in this figure are available as additional files Figure 5a Figure 5b, and Figure 5c.

Figure 5 shows examples of re-entry, wavebreak and propagation in the reference virtual tissue. In each case the pacing (S1), and premature (S2) stimuli were delivered to the bottom edge. In the top row (Figure 5a – see also the movie in additional file 1), the premature S2 activation was blocked at each of the regions with prolonged repolarisation, and curled round to give figure-of-8 re-entry. In the second row (Figure 5b – see also the movie in additional file 2) the S2 stimulus was 15 ms later, and although the S2 activation was partially blocked, re-entry was prevented by collision of the wavebreak with antegrade activation of the regions with prolonged APD. With a later S2 stimulus (Figure 5c – see also the movie in additional file 3), the regions with prolonged APD had recovered enough to conduct the S2 activation, although the activation wave was delayed slightly by each region with prolonged APD.

**Figure 6:**
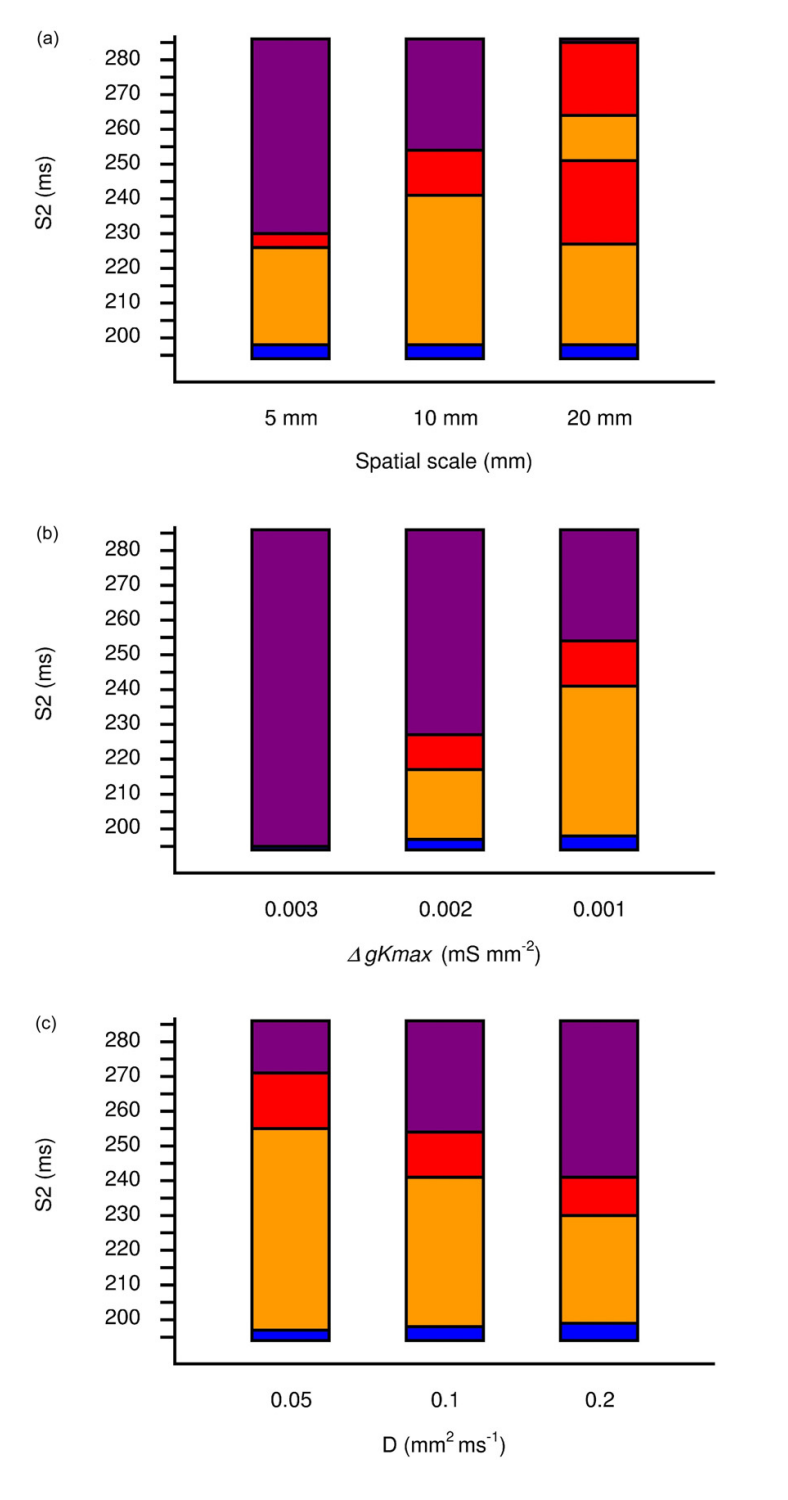
Response of virtual tissues to premature S2 stimulus. Blue indicates block, orange re-entry, red wavebreak, and purple propagation. (a) Results for changing in spatial scale, (b) changes in Δ*gKmax*, and (c) changes in strength of cell-to-cell coupling, where D is the diffusion coefficient.

Figure 6 shows the detailed response of each virtual tissue for a range of S2 intervals, and indicates how each of the three interventions affects the response of the tissue to a premature stimulus. Increasing spatial scale, increasing Δ*gkmax*, and decreasing the strength of cell to cell coupling all resulted in an greater range of S2 intervals that resulted in wavebreak (red) or re-entry (orange), and hence an increase in the width of the susceptible window The lower bound of the susceptible window where S2 was blocked depended on the refractory period of the border tissue with short APD, and was not greatly affected by changes in spatial scale, Δ*gkmax *or strength of cell-to-cell coupling. The upper bound showed a similar trend to the measures of APD dispersion shown in Figure 4, with a large spatial scale, large Δ*gkmax *and weak cell-to-cell coupling associated with a wide susceptible window.

**Figure 7:**
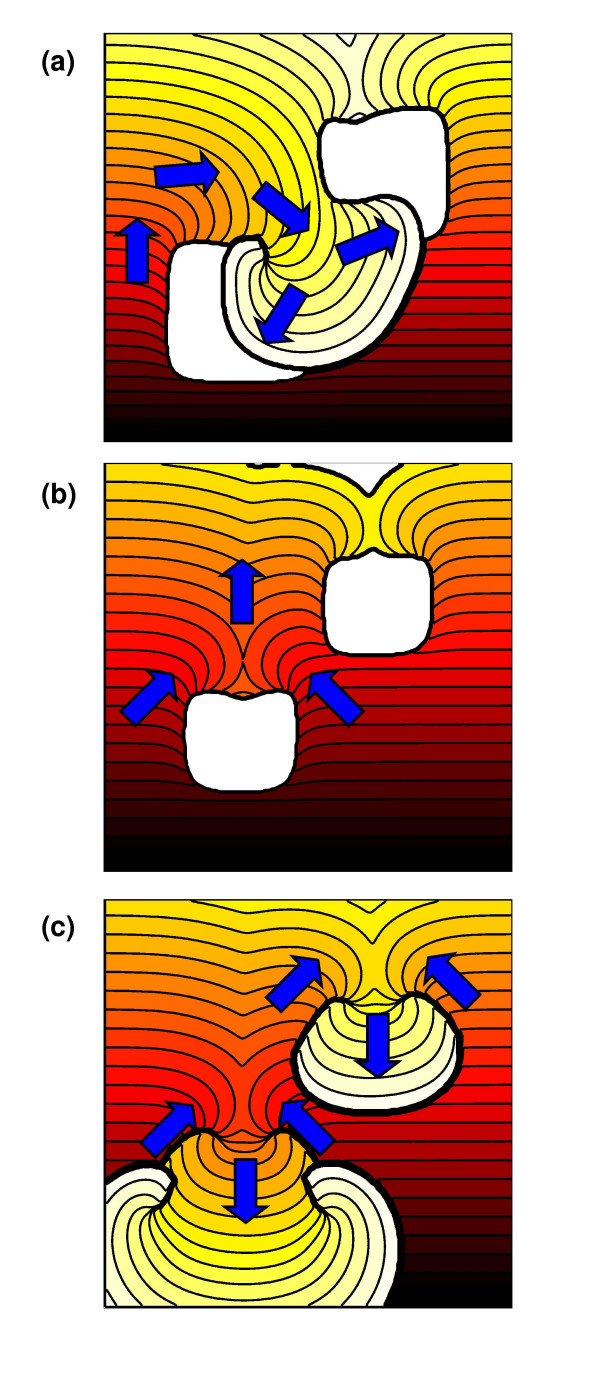
(See text for details) Example responses to premature S2 stimulus in virtual tissue with heterogeneity on spatial scale of 20 mm and Δ*gKmax *of 0.003 mS mm^-2^. Arrows show direction of propagation. (a) S2 at 200 ms and induction of re-entry with two phase singularities. (b) S2 at 245 ms and broken wave, retrograde activation is blocked. (c) S2 at 255 ms with retrograde activation and re-entry with four phase singularities *i.e. *two systems of figure-of-eight re-entry In each figure isochrones at intervals of 5 ms are shown, and the activation wavefront at S2+150 ms (a and c) and S2+155 ms (b) is shown as a thick black line. Movies of the simulations shown in this figure are available as additional files Figure 7a, Figure 7b, and Figure 7c.

The virtual tissue with a spatial scale of 20 mm showed a different pattern of susceptibility compared to the others, with two ranges of S2 that initiated wavebreak and two ranges of S2 that initiated re-entry. This behaviour is illustrated in Figure 7. For S2 delivered between 198 and 226 ms, the regions with prolonged APD blocked the premature activation, and re-entry was initiated by retrograde activation through the isthmus between these two regions (Figure 7a – see also the movie in additional file 4). For values of S2 between 227 and 249 ms, the isthmus conducted the S2 activation resulting in wavebreak, but the regions with prolonged APD remained refractory (Figure 7b – see also the movie in additional file 5). For values of S2 between 250 and 263 ms, re-entry was initiated by retrograde activation of the regions with prolonged APD (Figure 7c – see also the movie in additional file 6).

**Figure 8:**
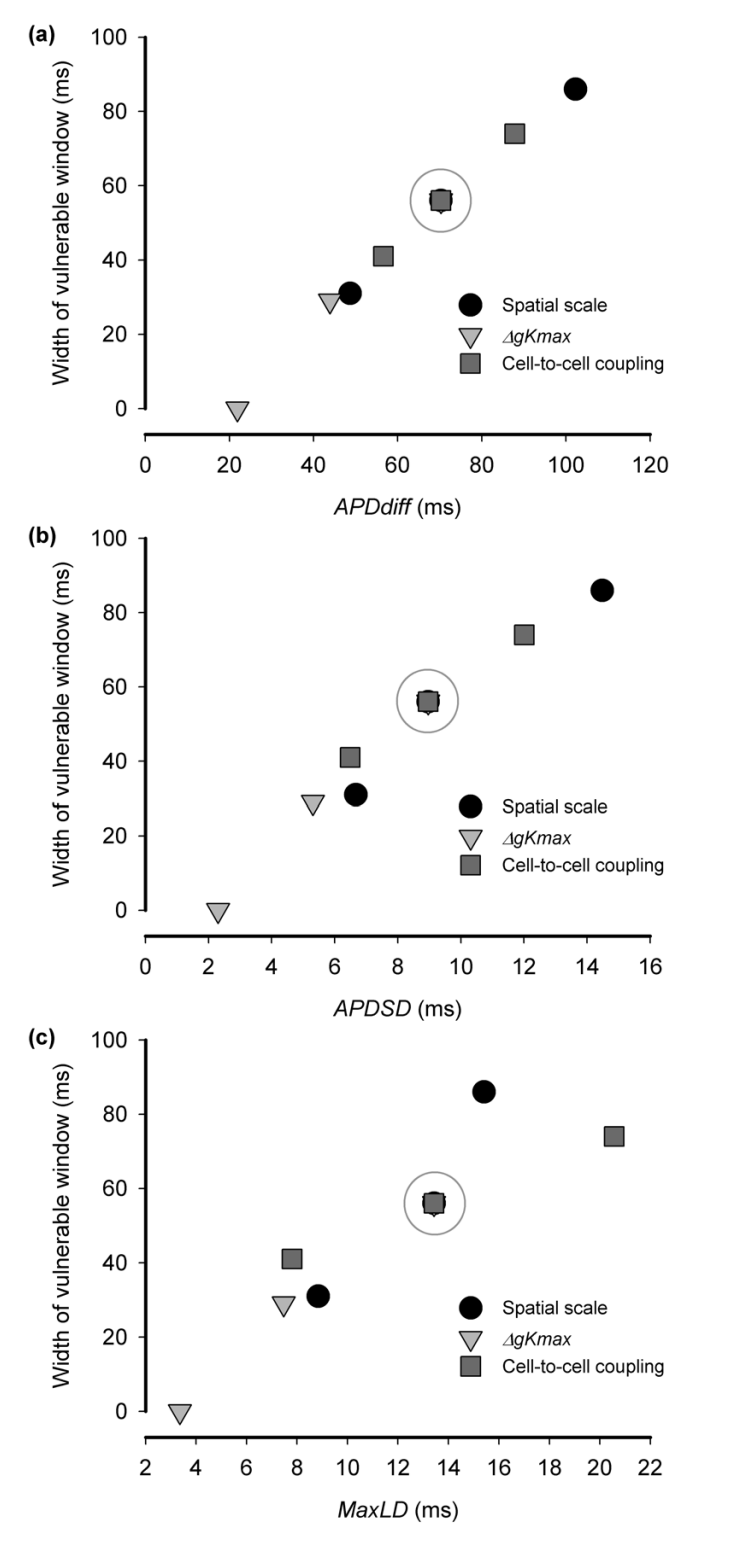
Association between width of the susceptible window and (a) *APDdiff*, (b) *APDSD*, and (c) *MaxLD*, for each of the three interventions In each case the grey circle indicates the reference virtual tissue with spatial scale of 10 mm, Δ*gKmax *of 0.003 mS mm^-2 ^and diffusion coefficient set to of 0.1 mm^2 ^ms^-1^.

Figure 8a shows the width of the susceptible window plotted against *APDdiff *and reveals an approximately linear relationship. The correlation coefficient R^2 ^= 0.99, which indicates a strong association between *APDdiff *and susceptibility. The interception of the line with the *APDdiff *axis also suggests that for the S1 S2 configuration used in this study, the susceptible window falls to zero for APD dispersion less than 20 ms. The association between the other measures of APD dispersion (*APDSD *figure 8b, *maxLD *Figure 8c) and susceptibility is also monotonic, but less well correlated than for *APDdiff*.

### 3.3 Potential antiarrhythmic strategies

Although prolonging τ_j _by a factor of 10 had only a small (< 2 ms) effect on maximum and minimum APD, the width of the susceptible window was decreased from 56 ms to 39 ms. The lower bound of the susceptible window moved from 198 to 216 ms, reflecting an increase in the refractory period of the virtual tissue as well as more prominent conduction velocity restitution.

Doubling *g*_*K*1 _increased current flow across the membrane during repolarisation, shortening APD by about 10% and decreasing *APDdiff *from 70 ms to 49 ms. The width of the susceptible window was also reduced from 56 ms to 42 ms when *g*_*K*1 _was doubled. The lower bound of the susceptible window moved from 198 ms to 175 ms, as a result of the shorter APD.

## 4. Discussion

In this study we have used a computational model of cardiac tissue to dissect out the effects of spatial scale, Δ*gkmax*, and strength of cell-to-cell coupling on APD dispersion and susceptibility to re-entry. Wavebreaks and re-entry can be created in cardiac tissue when an activation wavefront encounters a gradient of recovery. Experimental studies have therefore found an association between increased APD dispersion and greater susceptibility to re-entry because a premature stimulus is more likely to be blocked in tissue with regions of prolonged APD. In this study we found that large spatial scale heterogeneity, large Δ*gkmax*, and reduced strength of cell-to-cell coupling all increased both APD dispersion and susceptibility to re-entry. Tissue heterogeneities produce APD dispersion, and APD dispersion is modulated by electrotonic current flow [21]. In this study, spatial scale and Δ*gkmax *affected APD dispersion directly, whereas changing the strength of cell-to-cell coupling affected electrotonic current flow. This study indicates that each of these factors could be an important component in arrhythmogenesis, and that the susceptibility of a heterogenous tissue to re-entry can be estimated from simple measures of APD dispersion.

### 4.1 Relation to other work

Although the spatial scale of heterogeneity has been identified as potentially important in other studies [13], there is little information in the experimental literature to indicate how spatial scale affects susceptibility to re-entry. In one experimental study a ~1 cm2 region of thin layer of rabbit ventricular epicardium was cooled to produce a small region with prolonged APD, producing a dispersion of refractory periods ranging from 27 and 45 ms [10]. Re-entry could be initiated in this preparation using 4 increasingly premature stimuli. The spatial scale of heterogeneity in this experimental study was comparable to the reference virtual tissue used in our present study, but the effects of Δ*gkmax *and strength of cell-to-cell coupling in the experimental study are difficult to establish. Nevertheless the initiation of re-entry by block and retrograde activation in the region with prolonged APD followed a broadly similar pattern to our simulations, although the activation pathways in the experimental study were more complex, presumably due to anisotropic conduction in the rabbit ventricle. A decrease in strength of cell-to-cell coupling results in slowed conduction, and this is a common finding in tissue damaged by ischaemia, infarction [33], and other pathology [34]. Several studies have shown that decreasing cell-to-cell coupling can expose ionic heterogeneities [22,35-37].

Recent computational studies have addressed the influence of heterogeneous acetylcholine distribution on the vulnerability to and stability of re-entry in the atria [15,32]. The findings of these studies are broadly similar to the present study, although the effects of cell-to-cell coupling were not explicitly addressed, and initiation of re-entry was by either crossfield stimulation [32] or by S1 and S2 stimulation at different sites [15]. Both of these protocols would be expected to induce re-entry even in uniform tissue. In the present study both S1 and S2 stimuli were delivered from the same location, which does not initiate re-entry in uniform tissue, allowing us to examine the effect of heterogeneity on the initiation of re-entry in isolation.

The dynamical behaviour of APD is recognised as important not only for the stability of re-entry [2] but also in the development of alternans [38]. Recent experimental [39] and computational [38,40] studies have shown that APD dispersion can arise dynamically leading to discordant alternans, wavebreak, and re-entry in tissue that is either homogeneous or in which the ionic properties vary smoothly[38,41,42]. In the present study we measured APD dispersion at a fixed cycle length of 500 ms. The APD restitution curves given in Figure 1 indicate that APD dispersion could have been affected by pacing at shorter cycle lengths. This observation raises the possibility that heterogenous APD restitution could act to amplify APD dispersion.

The effect of heterogeneity on the stability of spiral waves has been investigated by Xie et al [43]. This study found that the amount of heterogeneity required to destabilise re-entry decreased as the degree of dynamical instability resulting from a steep APD restitution curve increased. In the present study we were interested in the initiation of re-entry rather than the stability of re-entry once initiated.

### 4.2 APD dispersion and susceptibility to arrhythmias

Normal ventricular tissue is remarkably resistant to the initiation of re-entry, but this robustness is greatly reduced by actions that increase the spatial dispersion of refractoriness. In this computational study we have shown that regional differences in repolarisation have an interlinked effect not only on the initiation of re-entry but also on measures of APD dispersion. Measures of APD dispersion are valuable in clinical practice because they could provide an estimate of arrhythmia risk, and various indices have been developed in experimental studies [17]. In our present study we have found that relatively simple measures of APD dispersion obtained from the tissue were related to the width of the susceptible period for re-entry.

### 4.3 Potential antiarrhythmic strategies

These preliminary investigations suggest that, in our model, susceptibility to re-entry could be reduced if recovery of Na^+ ^channels from inactivation can be prolonged, or if the conductance of the *i*_*K*1 _channel can be increased. The effect of this kind of intervention in the intact heart may however be more complex. Other computational studies have shown that modifying the kinetics of the Na^+ ^channel can have a pro-arrhythmic effect. Delaying recovery of Na^+ ^channels from inactivation can increase the slope of the APD restitution curve and hence the likelihood of alternans and re-entry [29], and reducing Na^+ ^channel conductance increases the vulnerable window [44]. Differences in the spatio-temporal complexity of VF between left and right ventricles have been attributed to differences in the current density of the *i*_*K*1 _channel in experimental studies [30]. Although this experimental finding is not directly connected to the effects of the *i*_*K*1 _channel conductance on susceptibility to re-entry investigated in the present study, it does highlight the potential importance of this channel for the mechanisms of re-entry.

The influence of individual ion channel currents on the initiation and subsequent behaviour of re-entry is an important direction for future research, but will require more biophysically detailed cell models than the LR1 model used in this study.

### 4.4 Limitations of the study

The electrical behaviour of cardiac tissue is complex, and depends on processes that act at tissue, cell, sub-cellular, and molecular levels. Computational models of electrical activation and conduction in the heart aim to simulate processes that are relevant to the research question, and simplifications are made accordingly. This study involved a large number of computations to establish susceptibility to re-entry, and so we chose to use a model that was a compromise between fidelity to real cardiac tissue and computational requirements.

More detailed versions of the LR model and others incorporating a fuller description of ion channels, pumps, exchangers, as well as Ca^2+ ^storage and release have been developed [22,25,45,46]. In tissue with regional ischaemia, Ca^2+ ^handling may become heterogeneous in addition to APD, and so it is possible that susceptibility to re-entry could also be modified if this additional feature is taken into account.

In the present study we chose to use an idealised geometrical heterogeneity based on square regions because this approach allowed us to assess the initiation of re-entry under well controlled conditions. In real cardiac tissue we would expect the heterogeneities to be much more irregular in shape and gradient, and the conditions that favour re-entry to be dependent on the relative location of the heterogenous region and the stimulus site.

The behaviour of re-entry in 3 D tissue is more complex than in 2 D, especially when the effects of rotational anisotropy and transmural differences in action potential shape and duration are taken into account [47]. Studies relating APD dispersion and susceptibility to re-entry in anatomically detailed 3 D tissue are another important project for the future. Recent computational studies indicate that the mechanical properties can not only modify the behaviour of re-entrant waves [48], but also that stretch activated channels in the cell membrane can contribute to susceptibility to re-entry if the tissue is stretched during repolarisation [49,50]. Since electrical repolarisation occurs at the same time as force generation in cardiac cells, the effect of cardiac mechanics on susceptibility to re-entry remains an important research question.

## Authors' contributions

**RHC **conceived and designed the study, wrote the simulation code, and ran the simulations. **AVH **participated in the study design, and helped draft the manuscript. Both authors read and approved the final manuscript.

## Supplementary Material

Additional File 1 Movie relating to Figure 5a Click here for file

Additional File 2 Movie relating to Figure 5b Click here for file

Additional File 3 Movie relating to Figure 5c Click here for file

Additional File 4 Movie relating to Figure 7a Click here for file

Additional File 5 Movie relating to Figure 7b Click here for file

Additional File 6 Movie relating to Figure 7c Click here for file
